# The diagnosis and treatment of retrograde intussusception: a single-centre experience

**DOI:** 10.1186/s12893-021-01391-0

**Published:** 2021-11-13

**Authors:** Bing Zhang, Dianming Wu, Mingkun Liu, Jianxi Bai, Fei Chen, Rong Zhang, Yifan Fang

**Affiliations:** Department of Pediatric Surgery, Fujian Maternity and Child Health Hospital, Affiliated Hospital of Fujian Medical University, 18 Daoshan Road, Gulou District, Fuzhou, 350001 Fujian China

**Keywords:** Anterograde, Retrograde, Intussusception, Children

## Abstract

**Background/Purpose:**

To investigate the clinical manifestations, treatments of retrograde intussusception and summarize the experience.

**Methods:**

Children with retrograde intussusception treated in our hospital from January 2011 to January 2021 were retrospectively analysed. Demographics, clinical manifestations, preoperative colour Doppler ultrasound (CDU) findings, findings during surgery and follow-up results were collected.

**Results:**

A total of 4719 cases of intussusception were treated in our department, including 12 cases of retrograde intussusception (0.25%). There were 8 males and 4 females.The age ranged from 4.1 to 14.3 months, with an average of (8.3 ± 2.8) months.; The weight ranged from 5.5 to 12.6 kg, with an average of (9.4 ± 2.3) kg; The onset time ranged from 6 to 15 h, with an average of (10.0 ± 2.4) h. All the children received CDU examination before surgery, and in one case, the possibility of 2 intussusception masses was considered. Emergency surgical exploration was performed after the failure of air enema reduction. During the operation, multiple types of intussusception were found (coincidence of anterograde and retrograde intussusception). The pattern of anterograde intussusception was all ileo-ileo-colic variety and the retrograde intussusception was proximal sigmoid colon into descending colon. All the children were successfully reduced by manual reduction without intestinal necrosis or intestinal malformation. All children were discharged 6–7 days after surgery, and had no recurrence after 3–6 months of follow-up.

**Conclusions:**

Retrograde intussusception is easily misdiagnosed before surgery. During air enema, if the intussusception mass was fixed and did not move with increasing pressure, we should be aware of the possibility of retrograde intussusception, and the enema pressure should not be too large to avoid intestinal perforation. If the intraoperative position of the intussusception mass was not consistent with that of the preoperative enema, it was recommended to use bimanual examination to explore whether there was still a mass in the abdominal cavity to avoid misdiagnosis.

## Introduction

Intussusception refers to intestinal obstruction caused by the insertion of a certain segment of the intestine and its corresponding mesentery into the adjacent intestinal cavity. It is the most common cause of intestinal obstruction in infants [1], with an overall incidence of approximately 18–8/100,000 [2]. Retrograde intussusception is a special type of intussusception, the incidence of this disease is extremely low. It is not easy to diagnose before surgery and is even difficult to find during surgery. This study retrospectively analysed a total of 12 cases of retrograde intussusception from January 2011 to January 2021, discussed and analysed the clinical manifestations, auxiliary examinations, and intraoperative exploration results of the children, and summarized the experience.

## Materials and methods

This study presents a retrospective review of the clinical data of paediatric patients with retrograde intussusception admitted to our department from January 2011 to January 2021. Demographic characteristics, medical history, clinical manifestations, preoperative colour Doppler ultrasound (CDU), air enema, intraoperative photos and follow-up results were collected. Inclusion criterion was: children with retrograde intussusception diagnosed during surgery. This study was approved by the Medical Ethics Committee of Fujian Maternal and Children’s Hospital, and the guardian of the child was informed about specimen collection procedures, examinations and project objectives; Informed consent forms were obtained for all patients.

## Results

A total of 4719 cases of intussusception were treated in our department during the study period, including 12 cases (8 males, and 4 females) of retrograde intussusception (0.25%). The age ranged from 4.1 to 14.3 months with an average of 8.3 ± 2.8 months; the weight ranged from 5.5 to 12.6 kg with an average of 9.4 ± 2.3 kg; the onset time ranged from 6 to 15 h with an average of 10.0 ± 2.4 h. Nine children were treated in the Paediatrics Department for diarrhoea during the first 3–5 days of onset. All the children had paroxysmal crying, nausea and vomiting, and 6 cases had jam-like bloody stools. A mass could be palpated in the upper abdomen of all the children. CDU examination revealed intussusception. In one case, two possible intussusception masses were considered. After obtaining the consent of the parents of the children, air enema was performed and the diagnosis of intussusception was confirmed. During the air enema, the pressure was started from 7.0 kPa. The intussusception mass was located at the proximal end of the sigmoid colon or the distal end of the descending colon. The pressure of the enema was gradually increased to 11.0 kPa, and the mass moved slightly to the proximal end, and stopped in the middle or distal ends of the descending colon. The intestinal tube was obviously dilated under fluoroscopy. The air enema was stopped, and emergency surgical exploration was planned. During the operation, multiple types of intussusception (coexistence of anterograde and retrograde intussusception) were found. The distal end of the anterograde intussusception mass was located near the ascending colon or proximal transverse colon, and the size was approximately 6 × 4 cm–15 cm × 5 cm. The proximal small intestine had dilatation and oedema, as well as gas and fluid accumulation in the intestinal cavity. The retrograde intussusception mass was located in the middle or proximal part of the descending colon, and the size was approximately 5 × 3 cm–7 × 4 cm. All the children were successfully reduced by manual reduction. The pattern of anterograde intussusception was all ileo-ileo-colic variety, the length of the ileum was approximately 10–25 cm. The retrograde intussusception was proximal sigmoid colon into descending colon (Table 1). None of the children had intestinal necrosis, intestinal malformation or space-occupying lesions (with the consent of the parents of the children, 6 children underwent intraoperative sigmoidoscopy). All children were discharged 6–7 days after the operation, and no recurrence was found at 3–6 months of follow-up.

**Table 1:** General information for the patients

Number	1 (Fig. 1)	2 (Fig. 2)	3	4 (Fig. 3)
Gender	Male	Male	Female	Male
Age (months)	4.1	6.4	7.8	14.3
Weight (kg)	5.5	8.3	9.3	12.6
Onset time (hours)	12	8	10.5	15
Operation time (min)	70	86	90	81
Length of stay(day)	7	7	7	6
Preoperative CDU	Intussusception mass	Intussusception mass	Intussusception mass	Two intussusception masses
Bloody stool	Yes	Yes	No	No
The tightness of retrograde intussusception	Not easy to reset	Easy to reset	Easy to reset	Not easy to reset
Position of intussusception mass	Ascending colon, descending colon	Left side of transverse colon, descending colon	Middle section of transverse colon, descending colon	Ascending colon, descending colon

Number	5	6	7	8
Gender	Male	Female	Male	Male
Age (months)	8.4	5.6	6.9	6.6
Weight (kg)	10.0	6.2	8.2	7.4
Onset time (hours)	12	6	10	8
Operation time (min)	74	86	90	88
Length of stay(day)	6	7	7	6
Preoperative CDU	Intussusception mass	Intussusception mass	Intussusception mass	Intussusception mass
Bloody stool	No	Yes	Yes	No
The tightness of retrograde intussusception	Easy to reset	Not easy to reset	Not easy to reset	Easy to reset
Position of intussusception mass	Middle section of transverse colon, descending colon	Ascending colon, descending colon	Ascending colon, descending colon	Left side of transverse colon, descending colon

Number	9	10	11	12
Gender	Female	Male	Female	Male
Age (months)	10.2	8.6	9.7	11.3
Weight (kg)	12.5	11.2	10.4	10.8
Onset time (hours)	10	8	11	9
Operation time (min)	72	90	77	84
Length of stay(day)	7	6	6	6
Preoperative CDU	Intussusception mass	Intussusception mass	Intussusception mass	Intussusception mass
Bloody stool	Yes	No	Yes	No
The tightness of retrograde intussusception	Easy to reset	Not easy to reset	Easy to reset	Not easy to reset
Position of intussusception mass	Left side of transverse colon, descending colon	Ascending colon, descending colon	Middle section of transverse colon, descending colon	Ascending colon, descending colon

## Discussion

Intussusception is mostly caused by intestinal peristalsis disorders, which may be related to the greater mobility of the ileocecal mesentery, adenovirus infection, hyperplasia of the lymphatic tissue in the distal ileum, and other causes of intestinal motility disorders, but also reported that bacterial enteritis is a risk factor [3]. According to the direction of intussusception, it can be divided into antegrade intussusception and retrograde intussusception. Anterograde intussusception is the invagination of the proximal part of the bowel into the lumen of the distal part, which occurs in most cases of intussusception. Retrograde intussusception refers to retrograde invagination of the distal segment of the intestine into the lumen of the proximal intestinal, and can be classified into jejunogastric intussusception, enteric intussusception, ceco-ileal intussusception or colic intussusception. Its causes are related to perverted peristalsis, which is a paralytic condition of the intestine and intestinal space occupying lesions [4]. The disease is quite rare, with an incidence of 0.2% [5], which is similar to this study. Most of the reports of the disease are jejunum-jejunum intussusception after obese patients underwent gastrectomy and gastrojejunostomy. The reason may be that the separation of the jejunum and duodenal pacemaker cells after surgery leads to abnormal peristaltic waves, the mesentery becomes thinner after weight loss and the jejunum-jejunum anastomosis is related [6]. It can also occur when a gastric catheter is placed after gastrostomy and when duodenal jejunostomy is performed for duodenal atresia [7, 8]. In this study, 9 children with retrograde intussusception had prior diarrhoea. It cannot be ruled out that diarrhea or the presence of antegrade intussusception induced abnormal peristalsis of the distal bowel, leading to the occurrence of retrograde intussusception. If no leading point is found after intraoperative reduction, only reduction can be performed without further treatment [9].

Multiple intussusceptions refer to the separation of two or more intussusceptions in different areas of the intestinal canal, which is relatively rare [10], and is mostly caused by intestinal intussusception or organic lesions [11]. In this study, there were two separate intussusceptions, antegrade intussusception and retrograde intussusception, and no space-occupying bowel lesions were found. Colocolic intussusception is relatively rare, with an incidence rate less than 5% of all cases of intussusception in children, most of which are case reports, and most of them are cases of anterograde intussusception caused by colon tumours or faecal stones in the colon [12–14], it may also be secondary to Crohn's disease [15]. The colocolic intussusception in this study was retrograde intussusception, and no tumours or faecal stones were found. Six children underwent colonoscopy and no abnormalities were found. All children in this study had typical paroxysmal crying, nausea, and vomiting. Six children had bloody stools, and the other 6 children did not. However, during the operation, it was found that the distal retrograde intussusception of the patient without bloody stool was tightly nested, and the typical jam-like bloody stool was discharged 2 h after the reduction. Therefore, we considered that these 6 patients had bloody stool before the operation. Because distal retrograde intussusception is too tight, it cannot be discharged. The order of occurrence of the two intussusceptions cannot be known, and all children had typical clinical manifestations of intussusception. So if retrograde intussusception occurs simply, whether there will be typical clinical manifestations is currently uncertain.

If intussusception is suspected, the preferred auxiliary examination is CDU. The sensitivity of CDU is 97.5% and its specificity is 99% [16]. There are also reports in the literature that multiple intestinal intussusceptions have been diagnosed by CDU [17]. Sanders et al. reported that computed tomography can be used for the diagnosis of antegrade intussusception, but for retrograde intussusception, the diagnosis is best confirmed with echocardiogram [18]. In this study, the abdominal mass could be palpated in all children, but we still improved the CDU examination. Only 1 case considered the possibility of multiple types of intussusception, but did not indicate whether it was retrograde intussusception. The volume of anterograde intussusception is generally larger than that of retrograde intussusception. If it is combined with retrograde intussusception, it should be found that the masses have changed from large to small during examination, which is inconsistent with typical anterograde intussusception. In this study, none of the children were considered to have retrograde intussusception before surgery. The cause may be that the doctor was not patient enough, and was only satisfied with the diagnosis of intussusception, without further confirming whether there was retrograde intussusception. When the mass of the intussusception is large, it is necessary to be aware of whether it is one mass or two close masses. It is recommended that the sonographer appropriately expand the range of CDU examination of the abdomen, and check from the upper rectum to the sigmoid colon to rule out retrograde intussusception.

Air enema or water enema under CDU monitoring is currently the preferred treatment method for intussusception reduction. However, for retrograde intussusception, whether it is an air enema or water enema, not only will it not reset the intussusception mass, but also will cause the mass of intussusception to become tighter as the enema pressure increases, and which may lead to intestinal perforation. In this study, with increasing enema pressure, there was no obvious reduction in the intussusception mass, and the diameter of the intestinal lumen increased. We considered the mass of the intussusception to be tight, and we did not think of the possibility of retrograde intussusception. Fortunately, the enema was stopped in time, and there was no intestinal perforation.

**Fig. 1:**
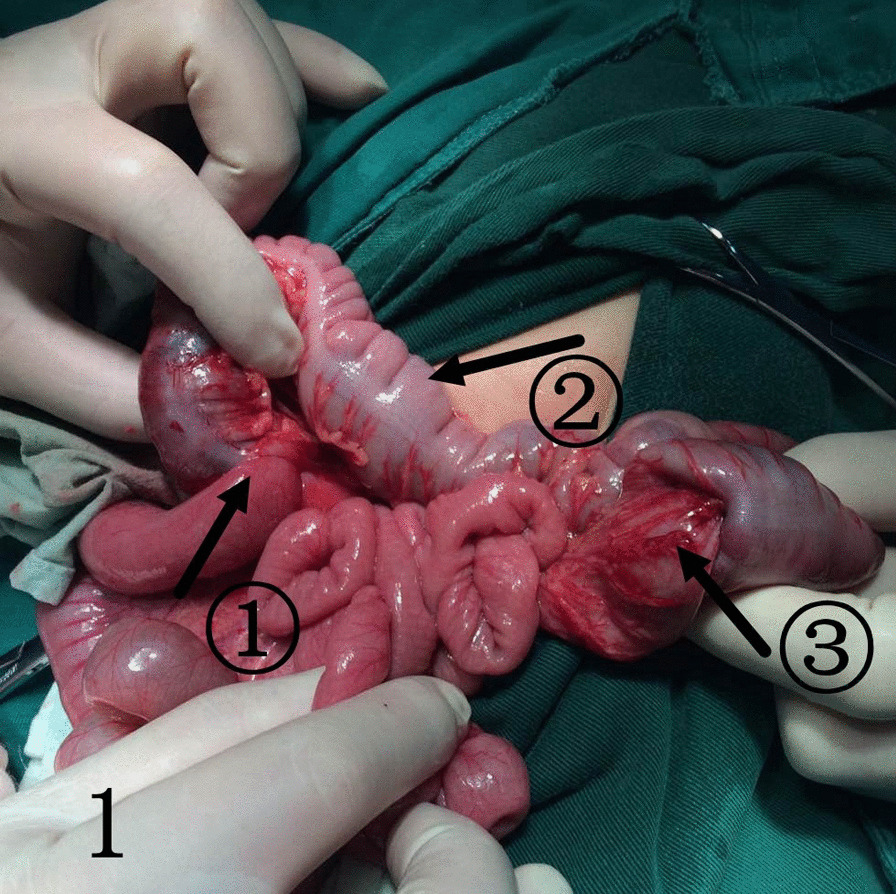
Indicated by the arrow: ①. antegrade intussusception; ②. transverse colon; ③. retrograde intussusception

**Fig. 2:**
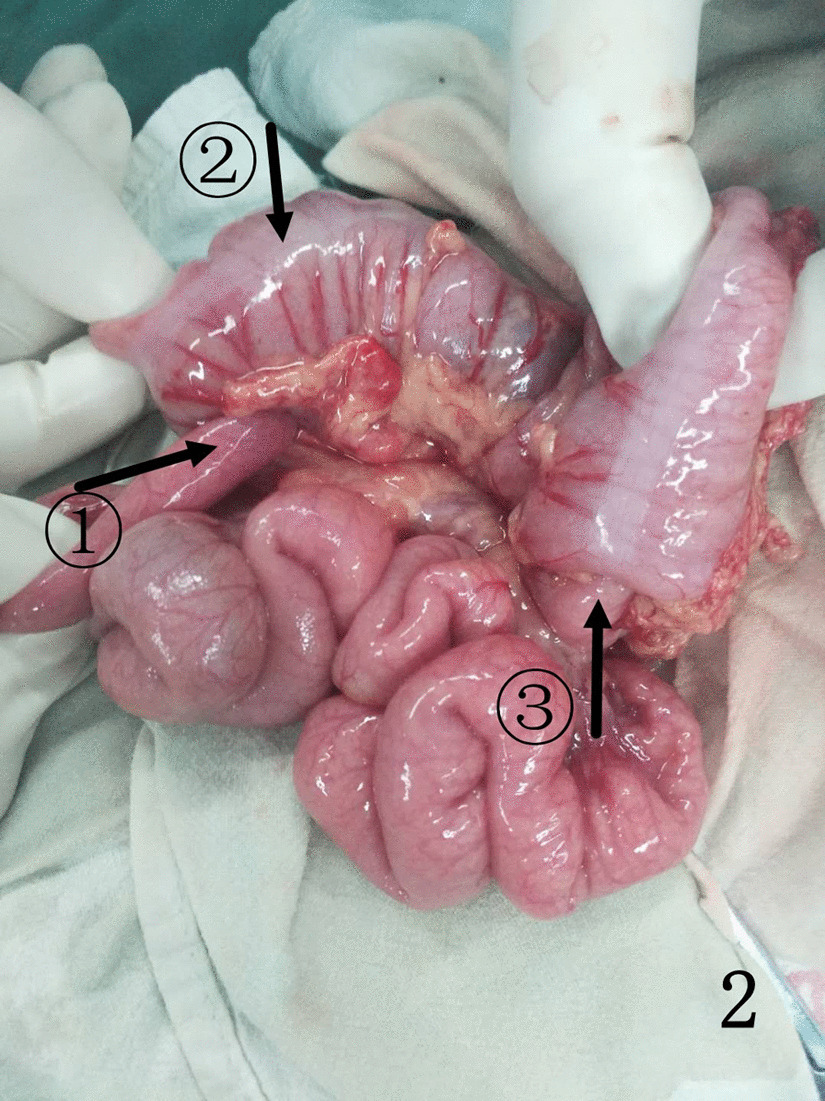
Indicated by the arrow: ①. antegrade intussusception; ②. transverse colon; ③. retrograde intussusception

**Fig. 3:**
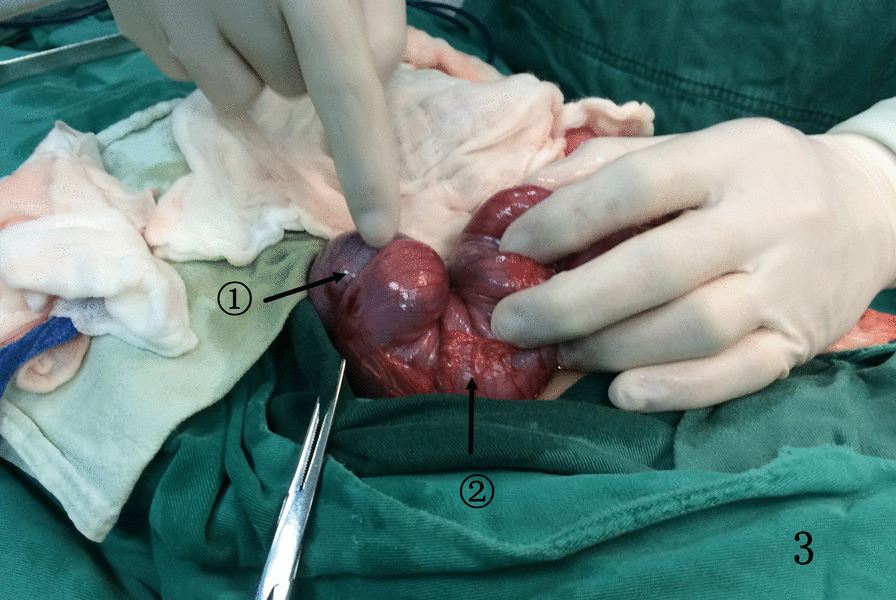
Indicated by the arrow: ①. retrograde intussusception; ②. transverse colon

For children with failed air enema reduction, surgery is the only option. The children in this study all underwent traditional open surgery. For the first child with retrograde intussusception, the intussusception mass was detected in the ascending colon during the operation, and the surgeon could easily lift the mass out of the incision. However, considering that the position of the intussusception mass was inconsistent with the preoperative air enema, the surgeon extended the index finger and middle finger of the right hand into the abdominal cavity, performed a bimanual examination with the left hand, and explored another mass in the lower left abdomen. After the incision was enlarged, the mass was dragged out of the incision, and it was found to be retrograde intussusception. Using this method, we found 11 more cases of retrograde intussusception. The retrograde intussusception mass will reset itself as the proximal bowel peristalsis increases and the pressure in the intestinal cavity increases, because the currently reported cases in the literature were all treated with surgery, so we cannot give a clear answer. However, in case 1 (Fig. 1), case 4 (Fig. 2), case (Fig. 3), case 7, case 10,and case 12, retrograde intussusception had obvious congestion and oedema, and the intussusception was tight. Intraoperative reduction required greater pressure, so the possibility of self-repositioning was lower. Egbuchulem et al. reported a case similar to ours, in which intestinal resection and anastomosis were performed due to distal retrograde intussusception of bowel necrosis [19]. Due to our timely discovery of retrograde intussusception, the possibility of reoperation in children was avoided. At present, it has been recommended to complete the intussusception reduction operation with the assistance of laparoscopy [20], so laparoscopy can be used for abdominal exploration to determine whether there are multiple types of intussusception or retrograde intussusception.

## Conclusions

The incidence of retrograde intussusception is extremely low, and it is easy to miss the diagnosis before surgery. Air enemas will only cause intussusception to be tighter and impossible to reset. Therefore, when it is found that the intussusception mass is fixed and does not move with the increasing pressure, in addition to considering the tightness of the intussusception mass, it is also necessary to be aware of the possibility of retrograde intussusception. The enema pressure should not be too high to avoid intestinal perforation. If the position of the intussusception mass is found to be inconsistent with that of the enema during the operation, it is recommended to use bimanual examination to explore whether there is a mass in the abdominal cavity to avoid a missed diagnosis.

## Data Availability

The datasets generated during and analysed during the current study are not publicly available due to the rules and regulations of our hospital but are available from the corresponding author on reasonable request.
